# Emergency Department demand associated with seasonal influenza, 2010 through 2014, New South Wales, Australia

**DOI:** 10.5365/wpsar.2017.8.2.002

**Published:** 2017-09-25

**Authors:** David J Muscatello, Kendall J Bein, Michael M Dinh

**Affiliations:** a School of Public Health and Community Medicine, University of New South Wales, Australia.; b Emergency Department, Royal Prince Alfred Hospital.; c Discipline of Emergency Medicine, The University of Sydney.

## Abstract

**Introduction:**

Influenza’s impact on health and health care is underestimated by influenza diagnoses recorded in health-care databases. We aimed to estimate total and non-admitted influenza-attributable hospital Emergency Department (ED) demand in New South Wales (NSW), Australia.

**Methods:**

We used generalized additive time series models to estimate the association between weekly counts of laboratory-confirmed influenza infections and weekly rates of total and non-admitted respiratory, infection, cardiovascular and all-cause ED visits in NSW, Australia for the period 2010 through 2014. Visit categories were based on the coded ED diagnosis or the free-text presenting problem if no diagnosis was recorded.

**Results:**

The estimated all-age, annual influenza-attributable respiratory, infection, cardiovascular and all-cause visit rates/100 000 population/year were, respectively, 120.6 (99.9% confidence interval [CI] 102.3 to 138.8), 79.7 (99.9% CI: 70.6 to 88.9), 14.0 (99.9% CI: 6.8 to 21.3) and 309.0 (99.9% CI: 208.0 to 410.1). Among respiratory visits, influenza-attributable rates were highest among < 5-year-olds and ≥ 85-year-olds. For infection and all-cause visits, rates were highest among children; cardiovascular rates did not vary significantly by age. Annual rates varied substantially by year and age group, and statistically significant associations were absent in several years or age groups. Of the respiratory visits, 73.4% did not require admission. The non-admitted proportion was higher for the other clinical categories. Around 1 in 100 total visits and more than 1 in 10 respiratory or infection visits were associated with influenza.

**Discussion:**

Influenza is associated with a substantial and annually varying burden of hospital-attended illness in NSW.

## Introduction

Influenza remains a public health challenge. (*1*) It is associated with annual, varying, excess deaths in populations internationally. (*2*, *3*) Influenza is a vaccine-preventable disease, (*1*) and the extent of its contribution to morbidity and mortality is poorly recognized. Estimating the burden of influenza in various settings is thus a priority for the World Health Organization (WHO). (*4*) There are few studies estimating the impact of influenza on lower severity health outcomes including hospital Emergency Department (ED) visits. (*5*-*8*) There is increasing recognition that the impact of influenza extends beyond respiratory illness to circulatory and other diseases. (*2*, *9*)

Influenza-related illness is poorly recorded in hospital and death databases, and counting only laboratory-confirmed influenza infections will markedly underestimate influenza’s population impact. Diagnoses commonly assigned to patients with an influenza infection in hospital EDs in Australia include fever, an unspecified infection or a non-respiratory illness. (*10*) During influenza season, febrile convulsions in infants increase. (*11*) Thus, statistical time-series analysis is used to estimate population levels of illness and death attributable to influenza. (*2*, *10*, *12*)

We used time-series analysis to estimate the rate, number and proportion of ED visits attributable to influenza in the state of New South Wales (NSW), Australia by age and year for the period 2010 to 2014. Since a proportion of visits lead to admission and can be included in hospitalization estimates, we also prepared estimates for non-admitted visits.

## Methods

### Study design and setting

This was a retrospective, ecological time-series analysis of ED visits recorded in a state-wide administrative information system database for NSW over the five calendar years of 2010 to 2014. NSW is the most populous state in Australia with a diverse urban and rural population of 7.5 million over 800 000 km^2^. (*13*, *14*)

### Data sources

The available study data included all visits recorded in the NSW Emergency Department Data Collection database during the study period. (*15*) The database contains routinely collected administrative and clinical data for patient-level visits across most public hospitals in NSW. Exclusion criteria were: hospitals not submitting data for the entire study period, prearranged (planned) visits that are usually for follow-up from a previous attendance, patients dead on arrival and transfers from other facilities. (*15*) Population denominators were obtained from the Australian Bureau of Statistics. (*14*)

Prior to analysis, we assigned a single, mutually exclusive clinical category to each visit in the ED database. The category was assigned using the recorded primary ED diagnosis code, and if necessary, the presenting problem, as defined in a previous publication, which also includes examples of diagnoses in each category. (*15*) For each ED visit, a physician selects a diagnosis name from a list used by the hospital’s electronic patient medical record information system. The information system then assigns a diagnosis code depending on the diagnostic classification standard used by the hospital. Classification standards vary among the information systems and can be: the International Classification of Diseases versions 9 or 10 (ICD-9, ICD-10) or the Systematized Nomenclature of Medicine Clinical Terminology (SNOMED-CT).

To estimate the contribution of influenza to population-level health outcomes, a time series representing the relative weekly change in incidence of influenza infections in the population is needed as an independent variable in the regression analysis. Since all influenza infections diagnosed in microbiology laboratories in Australia are notifiable to regional and state health authorities, we used influenza notifications from NSW to prepare the time series. Non-identified, state-level notification data are available from the national health authority. (*16*)

### Outcomes

We modelled outcome time series as population rates for each of respiratory, infection, cardiovascular and all-cause (total) visits. The association between influenza infection and adverse cardiovascular outcomes is becoming well known and is often included in influenza-attributable mortality studies. (*9*) All-cause visits cast the widest net to estimate total influenza-attributable outcomes in time-series approaches to influenza burden estimation. (*2*)

Time series of the total and non-admitted ED outcome rates were prepared for persons of all ages as well as age groups broadly consistent with WHO guidelines for influenza burden estimation: 0–4, 5–14, 15–49, 50–64, 65–84 and ≥ 85 years. (*4*)

### Analysis

We used semi-parametric generalized additive modelling to regress the ED outcome against the influenza time series. For each clinical category, age and admission status group, the weekly rates of ED visits provided the dependent (outcome) variable for a time-series model. Each time series included 260 observations covering whole weeks occurring during the study period.

Weekly influenza notification counts were split into separate variables for each year as there is an evident increase in influenza testing and thus notifications over time. (*2*, *17*) Each annual influenza time series was set to zero in all years except the year to which it referred.

Much of the variation in the observed time series of ED visits is not due to influenza and much of that variation may be due to seasonal and other nonlinear factors. Therefore, a natural cubic smoothing spline of time (represented by consecutive week number) was included in the model as a non-parametric independent variable. (*12*)

For a given clinical category, age group and admission status group, the model equation was:

**Figure:**
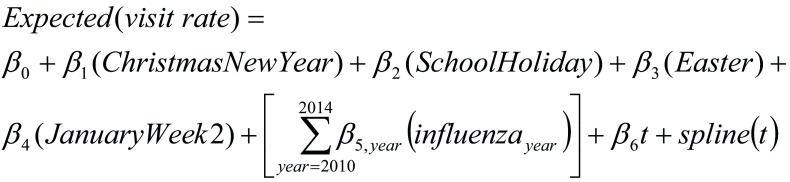


in which “Christmas,” “New Year,” “School Holiday,” “Easter” and “January Week 2” were holiday indicator variables (value 0 or 1) for periods of low ED demand, identified using a box whisker plot of the distribution of week of the year counts of all-cause visits. The *influenza_year_*variable was the respective annual weekly time series of seasonal influenza notifications. The *β* values were the model parameter estimates for the respective parametric independent variables, with *β*_0_**the model intercept. We specified 31 degrees of freedom for the flexibility of the smoothing spline based on previous research. (*12*)

The estimated weekly component of visits associated with the influenza variable was obtained by multiplying the influenza parameter estimate (*β*_5, _*_year_*) by the observed rate of the influenza variable (*influenza_year_*) in each week. Annual total counts were converted to rates using mid-year population estimates.

Estimated influenza-attributable counts and total counts, respectively, were used as the numerator and denominator for influenza-attributable proportions in each outcome category and age group.

Since estimates were made for numerous year, age group and visit category combinations, 99.9% confidence intervals (α = 0.001) were calculated to reduce chance statistical significance. We used the formula: parameter estimate ± 3.290 × standard error of the parameter estimate where 3.290 is the 99.9% critical value (z-value) from a standard normal distribution. Standard errors for confidence intervals of five-year averages were the square root of the sum of the squared standard errors of the annual values divided by five (the number of years averaged). Non-statistically significant annual values were included in averages as zero with zero standard error.

SAS version 9.4 (SAS, Cary, NC, USA) was used for analysis using procedures and options described elsewhere. (*12*) Normally distributed model residuals was assumed and this was checked using quantile-quantile (QQ) plots of the residuals. Lack of serial independence (autocorrelation) over time in the model residuals was checked using autocorrelation plots.

### Sensitivity analysis

As a sensitivity analysis to assess whether influenza incidence was associated with visit categories that would implausibly be caused by influenza, injury visit rates were also regressed on the influenza notification time series.

### Ethics

The study was approved by the NSW Population and Health Services Research Ethics Committee. Information that could identify patients was not included in the study data.

## Results

### Characteristics of the study data used

There were 11.8 million ED visits recorded between January 2010 and December 2014 of which 10.8 million visits to 115 hospitals met the inclusion criteria. Of these, 7.82 million (72.8%) were not admitted.

Among the clinical categories included in the study, injury comprised the largest group (mean = 117 visits/100 000 population/week) followed by respiratory (49.0 visits/100 000 population/week), cardiovascular (44.2 visits/100 000 population/week) and infection (22.9 visits/100 000 population/week). Among non-admitted visits, a similar pattern was observed. Increased influenza testing over time was evident; of the 44 308 influenza notifications during the study period, almost one half (20 744, 46.8%) occurred in 2014.

### Model fitting

Except for cardiovascular disease visits in the older population, at least one statistically significant holiday effect was identified in each clinical category and age group. Apart from some departures from normality for extreme observations and some residual autocorrelation, the QQ plots showed the modelling provided a good fit to the observed data.

### Main results

**Fig. 1:**
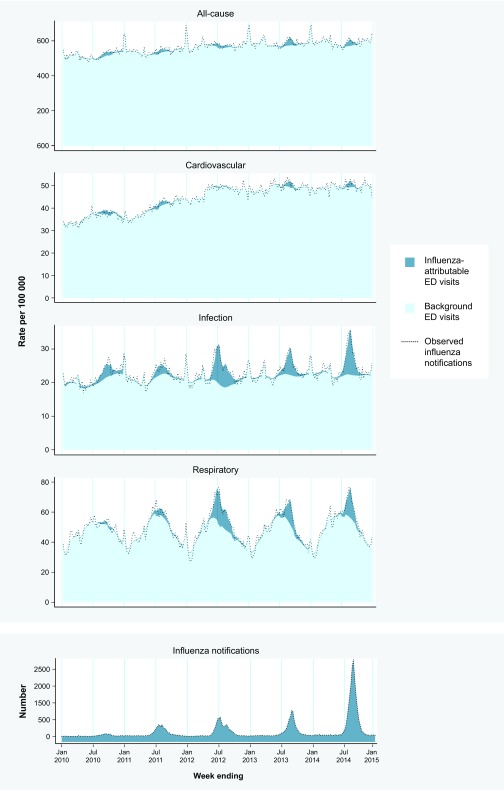
Observed weekly counts of influenza notifications, estimated influenza-attributable and non-influenza-attributable (background) ED visit rates/100 000 population/week for all-cause, cardiovascular, infection and respiratory clinical categories and observed visit rates/100 000 population/week in each clinical category, for persons of all ages, NSW, 2010 through 2014 (*n* = 260 weeks)

Exceedances in visit rates associated with circulating influenza are evident in each clinical category, particularly respiratory and infection. The exceedances are most distinct in years 2012, 2013 and 2014 (**Fig. 1**).

For respiratory visits, exceedances were greatest in 2012 in < 5-year-olds (939.0 visits [99.9% confidence interval (CI) 559.4 to 1318.7]/100 000 population/year) and ≥ 85-year-olds (987.7 [99.9% CI: 793.4 to 1181.9]/100 000 population/year) (**Fig. S1**, **Table S1**, **Supplementary File 1**). For infection visits, exceedances were most prominent in < 5-year-olds in 2012 (821.6 visits [99.9% CI: 657.8 to 985.4]/100 000 population/year) (**Fig. S2**, **Table S1**). Exceedances among cardiovascular visits were more difficult to distinguish and were most evident in ≥ 85-year-olds in 2011 (433.0 [99.9% CI: 179.8 to 686.3]/100 000 population/year) (**Fig. S3**, **Table S1**). Among all-cause visits, 2012 again stood out in < 5-year-olds (2368.0 [99.9% CI: 1544.1 to 3191.8]/100 000 population/year) and ≥ 85-year-olds (1778.4 [99.9% CI: 1060.0 to 2496.9]/100 000 population/year) (**Fig. S1**, **Table S1**).

When averaged from 2010 through 2014, there was a U-shaped relationship between age and estimated respiratory visit rates with < 5-year-olds and ≥ 65-year-olds higher than 15–64-year-olds based on confidence intervals. Compared with other age groups, estimated infection visit rates were highest in < 5-year-olds, followed by 5–14-year-olds, and these differences were statistically significant. The estimated infection visit rate in < 5-year-olds was significantly higher than the estimated respiratory rate in the same age group. For cardiovascular visits, significant rates were present only in the 5–14, 15–49 and 65–84 age groups, and these were not significantly different across those age groups. Estimated all-cause rates were highest in < 5-year-olds and declined with age until the 50–64 year age group and then increased again with the rate in ≥ 85-year-olds about half that of < 5-year-olds (**Table 1**).

**Table 1:** Average annual estimated rate/100 000 population and number of influenza-attributable ED visits, by clinical category and age, NSW, 2010 through 2014

Clinical category	Age group (years)	Rate	(99.9% CI)	Number	(99.9% CI)
Respiratory	0–4	187.8	(111.9 to 263.7)	902	(537 to 1 266)
	5–14	106.9	(71.2 to 142.6)	976	(651 to 1 301)
	15–49	80.7	(72.6 to 88.9)	2845	(2 558 to 3 132)
	50–64	95.0	(80.7 to 109.3)	1275	(1 085 to 1 465)
	65–84	178.6	(160.1 to 197.1)	1724	(1 544 to 1903)
	≥ 85	208.6	(115.1 to 302.0)	335	(200 to 470)
	All ages	120.6	(102.3 to 138.8)	8887	(7 548 to 10 227)
Infection	0–4	361.0	(291.2 to 430.8)	1740	(1 405 to 2076)
	5–14	144.1	(123.3 to 165.0)	1304	(1 117 to 1 492)
	15–49	57.9	(51.2 to 64.6)	2035	(1 800 to 2 269)
	50–64	24.8	(20.1 to 29.6)	336	(272 to 401)
	65–84	25.6	(19.7 to 31.6)	248	(191 to 305)
	≥ 85	45.5	(25.2 to 65.8)	68	(38 to 99)
	All ages	79.7	(70.6 to 88.9)	5856	(5 192 to 6 519)
Cardiovascular	0–4	*-3.4*	*(−5.8 to −0.9)*	*-16*	*(−28 to −4)*
	5–14	6.3	(2.8 to 9.7)	57	(26 to 89)
	15–49	5.7	(1.9 to 9.6)	203	(67 to 339)
	50–64	0.0	(0.0 to 0.0)	0	(0 to 0)
	65–84	16.7	(0.2 to 33.2)	152	(2 to 301)
	≥ 85	17.0	(−63.3 to 97.3)	28	(−82 to 138)
	All ages	14.0	(6.8 to 21.3)	1033	(499 to 1 567)
All-cause	0–4	1013.9	(692.1 to 1 335.8)	4856	(3 315 to 6 397)
	5–14	455.5	(311.7 to 599.3)	4115	(2 820 to 5 410)
	15–49	175.3	(102.8 to 247.7)	6174	(3 625 to 8 724)
	50–64	49.3	(15.7 to 82.9)	668	(213 to 1 123)
	65–84	194.3	(125.0 to 263.6)	1870	(1 199 to 2 540)
	≥ 85	506.9	(318.4 to 695.5)	761	(475 to 1 047)
	All ages	309.0	(208.0 to 410.1)	22 619	(15 268 to 29 969)

Notes: Non-statistically significant annual values were included in averages as zero, with zero standard error. CI = confidence interval. Statistically significant positive results are shown in bold. Statistically significant negative results are italicized.

Among persons of all ages, the average annual estimated influenza-attributable rate for respiratory visits was 120.6 [99.9% CI: 102.3 to 138.8]/100 000 population/year (8887 [99.9% CI: 7548 to 10 227] visits/year). For infection visits, the rate was 79.7 [99.9% CI: 70.6 to 88.9]/100 000 population/year (5856 [99.9% CI: 5192 to 6519] visits/year). For cardiovascular visits, the average was 14.0 [99.9% CI: 6.8 to 2.3]/100 000 population/year (1033 [99.9% CI: 499 to 1567] visits/year). For all-cause visits, the all-age average annual estimated rate was 309.0 [99.9% CI: 208.0 to 410.1]/100 000 population/year (22 619 [99.9% CI: 15 268 to 29 969] visits/year) (**Table 1**).

In < 50-year-olds, differences between the estimated rates in total and non-admitted visits are not significantly different. In older age groups, the rates of estimated non-admitted visits were substantially and significantly lower than those of total visits, particularly for the respiratory and infection categories. For persons of all ages, estimates for non-admitted visits were broadly similar to those of total visits. Averaged across all years, these patterns were also broadly reflected. These results indicate that older persons with influenza-related illness are more likely to be admitted (**Tables S2** and **S4**, **Supplementary File 1**).

Compared with total visits (**Table 1**), a similar pattern of statistically significant associations was evident among non-admitted visits. The average annual estimated all-age rate of influenza-attributable, non-admitted respiratory visits was 88.5 (99.9% CI: 74.7 to 102.3)/100 000 population/year or 73.4% of total influenza-attributable respiratory visits/year). For non-admitted infection visits, the rate was 69.8 (99.9% CI: 61.9 to 77.7)/100 000 population/year (87.4%). For non-admitted cardiovascular visits, the rate was 12.2 (99.9% CI: 7.9 to 16.5)/100 000 population/year (87.1%). The rate of excess average annual all-cause non-admitted visits was 287.1 (99.9% CI: 196.2 to 378.0)/100 000 population/year; 92.8%) (**Table 2**).

**Table 2:** Average annual estimated rate/100 000 population and number of influenza-attributable *non-admitted* ED visits, by clinical category and age, NSW, 2010 through 2014

Clinical category	Age group (years)	Rate	(99.9% CI)	Number	(99.9% CI)
Respiratory	0–4	276.8	(185.9 to 367.8)	1337	(896 to 1 778)
	5–14	142.7	(106.5 to 179.0)	1295	(968 to 1 621)
	15–49	69.0	(61.9 to 76.2)	2432	(2 182 to 2 682)
	50–64	54.2	(44.9 to 63.4)	727	(605 to 850)
	65–84	70.6	(59.9 to 81.4)	682	(578 to 787)
	≥ 85	30.6	(2.4 to 58.8)	49	(8 to 91)
	All ages	88.5	(74.7 to 102.3)	6525	(5 511 to 7 538)
Infection	0–4	319.5	(258.2 to 380.7)	1539	(1 245 to 1 833)
	5–14	131.6	(112.8 to 150.5)	1191	(1 021 to 1 361)
	15–49	53.3	(47.7 to 59.0)	1875	(1 677 to 2073)
	50–64	18.5	(14.9 to 22.2)	251	(202 to 300)
	65–84	11.9	(7.1 to 16.6)	113	(69 to 158)
	≥ 85	*-15.3*	*(−22.8 to −7.7)*	*-22*	*(−32 to −11)*
	All ages	69.8	(61.9 to 77.7)	5121	(4 547 to 5 694)
Cardiovascular	0–4	*-2.2*	*(−4.2 to −0.2)*	*-11*	*(−21 to −1)*
	5–14	5.8	(2.7 to 8.9)	53	(25 to 82)
	15–49	9.3	(5.4 to 13.2)	329	(191 to 467)
	50–64	5.6	(1.3 to 9.9)	77	(17 to 136)
	65–84	12.8	(4.2 to 21.4)	116	(38 to 194)
	≥ 85	22.0	(−6.2 to 50.3)	30	(−11 to 70)
	All ages	12.2	(7.9 to 16.5)	900	(586 to 1 214)
All-cause	0–4	1090.0	(787.1 to 1 392.8)	5235	(3 780 to 6 690)
	5–14	432.0	(301.3 to 562.7)	3903	(2 726 to 5 080)
	15–49	174.2	(109.7 to 238.8)	6141	(3 869 to 8 413)
	50–64	89.4	(37.5 to 141.3)	1177	(497 to 1 857)
	65–84	122.3	(52.1 to 192.4)	1106	(473 to 1 739)
	≥ 85	*-93.9*	*(−178.7 to −9.1)*	*-132*	*(−252 to −13)*
	All ages	287.1	(196.2 to 378.0)	20 997	(14 383 to 27 611)

Notes: Non-statistically significant annual values were included in averages as zero, with zero standard error. CI = confidence interval. Statistically significant positive results are shown in bold. Statistically significant negative results are italicised.

An annual average of 4.7% of total respiratory visits and 5.6% of non-admitted respiratory visits was estimated to be attributable to influenza. Among infection visits, the average annual proportion was 6.7% (non-admitted: 8.4%). Among cardiovascular and all-cause visits, the average annual proportions were 0.6% (non-admitted: 1.1%) and 1.1% (non-admitted: 1.3%), respectively. The highest proportion by age was 12.4% (non-admitted: 13.6%) of infection visits in 5–14-year-olds (**Table S5**, **Supplementary File 1**).

### Sensitivity analysis

When injury visit rates were regressed on influenza incidence, a positive statistically significant association was identified for one age group in one year (5–14 year-olds in 2013; 208.5 (99.9% CI: 44.9 to 372.2)/100 000 population/year). Statistically significant negative results were identified for six of seven age groups in 2012 and three of seven age groups in 2014 (**Table S6**, **Supplementary File 1**). The overall proportion of estimated influenza-attributable injury visits was −0.6%.

## Discussion

We estimated that influenza was associated with approximately 1 in every 100 ED visits and more than 1 in 10 respiratory or infection visits, on average, concentrated across mid-winter to early spring. Over 300 all-cause visits/100 000 population/year were associated with influenza. Of these, 121 and 80/100 000 population/year were respiratory and infection visits, respectively. Influenza possibly explained 14 cardiovascular visits/100 000 population/year, although age groups and years with significant associations did not appear comparable with those for respiratory and infection visits. Depending on the type of visit, the burden appears to be borne to the greatest degree by the youngest and oldest age groups. Over 1000 all-cause visits/100 000 population/year in children aged under 5 years were associated with influenza and over 400/100 000 population/year in ≥ 85 year-olds. Approximately three quarters of influenza-attributable respiratory visits did not require admission, compared with 87% for infection visits and 93% of all-cause visits. Young children were less likely than older adults to be admitted to hospital.

Our post-pandemic, influenza-attributable, annual respiratory visit rate estimate of 121/100 000 population/year for the period 2010–2014 in NSW was substantially lower than estimates from Ontario, Canada and New York, NY, USA at different time periods. (*5*, *8*) Varying influenza activity and virulence over time, immunization coverage and effectiveness, availability and cost of health services or different modelling approaches could explain the difference. The post-pandemic year in our study, namely 2010, did have unusually low influenza activity, and our modelling approach may provide more conservative estimates than the modelling method used in the other studies. (*12*)

Interpreting variation in influenza-attributable burden from year to year requires an understanding of the influenza strains that circulated and the effectiveness of influenza vaccines. In Australia, influenza vaccination is free to certain risk groups, the largest group being the older population, with coverage around 70% in ≥ 65-year-olds during the study period. (*18*) Coverage in younger persons is substantially lower: around 33% in 50–64-year-olds and below 20% in younger age groups. (*19*)

The 2009 influenza A(H1N1)pdm09 pandemic strain dominated in Australia in 2010, although levels of circulation were low (**Table S7**, **Supplementary File 1**). This is consistent with the low levels of influenza-attributable ED demand, although there was substantial demand in the younger age groups, particularly in the infection clinical category.

Influenza A(H3N2) reappeared in 2011 and co-circulated with the pandemic strain, but overall levels remained relatively low (**Table S7**). The vaccine in those years showed good effectiveness of at least 70% against the pandemic strain. (*20*, *21*) The older age group experienced relatively low susceptibility to the pandemic strain due to pre-existing immunity. (*2*, *12*, *19*, *22*, *23*) These combined factors would explain the relatively low overall levels of influenza-attributable visits in 2010–2011.

The 2012 season showed the highest relative influenza circulation of the study period with A(H3N2) dominating and influenza B accounting for the remainder (**Table S7**). This season had the highest estimated incidence of respiratory or infection ED visits of all years studied in persons of all ages and in < 5-year-olds. The rates in ≥ 65-year-olds were also highest in 2012 for respiratory, infection and all-cause visits. Vaccine effectiveness in Australia against the circulating H3N2 strain in that season was low at 30% in 2012, which may be due to antigenic drift in the H3N2 virus. Effectiveness against influenza B was moderate (56%). (*24*)

There was relatively low overall influenza circulation in 2013. Influenza A(H3N2) continued to dominate (**Table S7**). Substantial estimated influenza-attributable visits were evident in younger age groups broadly comparable to surrounding years. Vaccine effectiveness against A(H3N2) in 2013 was good at 67%, (*24*) which may explain the relatively lower levels of influenza-attributable visit rates in older age groups compared with the surrounding years.

In 2014, the pandemic strain dominated but A(H3N2) co-circulated. This year had the second highest total apparent influenza circulation of the years studied (**Table S7**). In that year, the vaccine effectiveness was moderate (55%) against pandemic A(H1N1) but low (26%) against A(H3N2), (*24*) possibly explaining substantial influenza-attributable rates in the older population.

The study had limitations. In NSW, the ED diagnosis is recorded as part of the routine workflow of physicians and not by health information managers trained in health care classification. Changes in information systems over time may have led to inconsistencies in the recording of diagnoses and other information. Diagnosis classifications varied across hospitals. (*15*) We did not have information on other viruses, such as respiratory syncytial virus, which may have caused some residual confounding. Biases can arise in the influenza notifications we used because the decision to test for influenza is at the discretion of the health-care provider and notifications arise from any type of medical service. On the other hand, notifications provide a combination of wide geographic coverage and are very specific to influenza.

Of the 186 EDs in NSW, 19% did not report to the Emergency Department Data Collection database during the study period and another 3% were excluded. Therefore, our results will be an underestimate of state-wide figures. Non-participating and excluded hospitals were smaller regional hospitals in more remote areas. (*15*) Nevertheless, the data set we analysed included approximately 87% of public hospital visits in the state. (*25*) ED services in NSW are almost wholly public. (*26*, *27*)

The statistically significant associations with injury are challenging to explain. The positive association may have been a chance finding even though we set a restrictive level of statistical significance. Unmeasured confounding could have occurred due, for example, to weather or other factors that vary on a similar time scale to influenza seasons. Confinement at home due to influenza may lead to fewer opportunities for injury, although this needs further study.

Using all-age rather than age-specific influenza notifications in the model might explain negative influenza-attributable estimates, which are, in reality, impossible. Using age-specific notifications in the model may improve estimates, but this requires further research.

In summary, seasonal influenza is associated with a substantial but annually varying burden of hospital-attended illness on EDs in NSW and thus on the overall population. The greatest demand occurs among young children and in the oldest population in some years. Varying vaccine effectiveness may have explained varying impact in the relatively well immunised older population. Improved vaccines and vaccination strategies that protect young children as well as older adults are needed to reduce morbidity in the population. Influenza surveillance information may be useful in forecasting and managing peaks in ED demand and to facilitate improved workload, staff and bed management. Improved control of influenza may substantially reduce surges in ED demand caused by influenza.
